# The frequency of microscopic and focal active colitis in patients with irritable bowel syndrome

**DOI:** 10.1186/1471-230X-11-96

**Published:** 2011-08-31

**Authors:** Kamil Ozdil, Abdurrahman Sahin, Turan Calhan, Resul Kahraman, Adil Nigdelioglu, Umit Akyuz, Hacı M Sokmen

**Affiliations:** 1 Department of Gastroenterology, Umraniye Training and Research Hospital, Adem Yavuz street No:1, Umraniye, Postal code:34766, Istanbul, TURKEY; 2 Department of Gastroenterology, Fatih Sultan Mehmet Training and Research Hospital, Ataşehir, Postal code: 34770, Istanbul, TURKEY

**Keywords:** irritable bowel syndrome, microscopic colitis, focal active colitis

## Abstract

**Background:**

Irritable bowel syndrome (IBS) is a chronic functional bowel disorder. The frequency of microscopic colitis and focal active colitis in the colonic mucosa has been investigated in IBS patients.

**Methods:**

Between June 2007 and September 2010, 378 patients (between 16 and 84 years) were recruited prospectively. Of these 378 patients, 226 patients were diagnosed with IBS using the Rome III criteria. 152 control patients were also enrolled who were undergoing colonoscopy for colorectal cancer screening or investigation of anemia. Histopathological abnormalities identified during colonoscopy were compared between the IBS and control groups.

**Results:**

The average age of the IBS group was 46.13 ± 14.16 years and and the average age of the control group was 57.01 ± 13.07 years. The prevalence of microscopic colitis (MC) in the diarrhea predominant and the mixed subgroup of IBS patients was 4.32% (7/162) whereas in all IBS patients, the prevalence was 3.09% (7/226). MC was not found in the 152 control cases, (p = 0.045). Lymphocytic colitis was seen in 7 IBS patients, with 1 case in the mixed group and 6 cases in the diarrhea group and there was a significant difference in the frequency of lymphocytic colitis between the IBS subgroups (p < 0.01). Focal active colitis was found in 6.6% (15/226) of the IBS patients and in none of the controls (p < 0.01), and there was no differences between IBS subtypes.

**Conclusion:**

Microscopic colitis was more often found in the diarrhea predominant/mixed subgroups of IBS patients and in patients who were older women. In patients who are older woman with non-constipated IBS, it may be reasonable to perform a biopsy to screen for microscopic colitis. Focal active colitis was significantly increased in patients with IBS compared to controls.

## Background

Irritable bowel syndrome (IBS) is a functional bowel disorder of unknown etiology without a curative treatment. In addition to abdominal discomfort and pain, relief from discomfort upon defecation and/or abdominal pain is associated with a change in the frequency of defecation and/or in the form of the feces [1]. The prevalence of IBS varies in different populations. The prevalence of IBS in Turkey was found 7.4-19.1% with female percentage between 64% and 69% [2,3]. Similar rates have been reported in the USA and Europe and range between 6.2% and 25% [4,5]. Although the specific location and the pattern of symptoms varies among different functional gastrointestinal diseases, there are also many common characteristics [6]. A small percentage of patients with IBS are referred to outpatient gastroenterology clinics.

In 1980, microscopic colitis (MC) was defined as having symptoms of watery diarrhea and specific histological characteristics when the colonic mucosa is macroscopically normal or near normal [7]. Currently, MC is divided into two subtypes- collagenous colitis (CC) involving chronic mucosal inflammation and a wide subepithelial band and lymphocytic colitis (LC) involving chronic mucosal inflammation and no subepithelial band. In 1976, Lindstrom showed for the first time that collagen deposits accumulated as a wide subepithelial band in the rectum and colon in patients with persistent watery diarrhea of unknown cause [8]. Some experts claim that LC is an early phase of CC [9-11]. In Europe, the annual incidence of CC is 0.6-2.3/100000 and the prevalence is 10-15.7/100000 whereas the annual incidence of LC is 3.1/100000 and the prevalence is 14.4/100000 [12-16]. MC and IBS have similar symptoms. MC not only leads to diarrhea but also causes constipation. Constipation may be short-term or chronic [17,18].

Focal active colitis (FAC) is characterized by focal crypt damage caused by neutrophils and may be associated with infections, ischemia, Crohn's disease, partially-treated ulcerative colitis and IBS [19]. It may consist of one focus that can be detected in a single biopsy, or multiple foci. Since focal inflammation is a feature of Crohn's disease, studies of FAC have examined whether it precedes the subsequent development or diagnosis of Crohn's disease. In two studies of adult patients with FAC, it was observed that very few developed Crohn's disease [19,20]. However, in a group of pediatric patients with FAC, a higher proportion (27.6%) went on to develop Crohn's disease [21].

The purpose of this study was to determine histopathologically the frequency of underlying organic causes of IBS, including MC and FAC, in macroscopically normal colonic mucosa.

## Methods

Between June 2007 and September 2010, 378 patients (between 16 and 84 years) were recruited into the study prospectively. Two hundred and twenty six of these patients were diagnosed with IBS using the Rome III criteria and had no alarm symptoms, no chronic disease history and had macroscopically normal colonoscopy/upper gastrointestinal system endoscopic findings. There were 152 control patients who had no history of chronic disease or drug use, who were undergoing colonoscopy for reasons not related to IBS (familial colorectal cancer screening or investigation of anemia) and who had macroscopically normal colonic mucosa.

Patients with IBS were divided into 3 subgroups according to the Rome III criteria. Patients with hard or lumpy stools ≥ 25% and loose (mushy) or watery stools < 25% of bowel movements the constipation-predominant group (IBS-C). Patients with loose (mushy) or watery stools ≥ 25% and hard or lumpy stools < 25% of bowel movements as the diarrhea-predominant group (IBS-D). Patients expressing hard or lumpy stools ≥ 25% and loose (mushy) or watery stools ***≥ ***25% of bowel movements were defined as the mixed group (IBS-M) [22-24]. The presence of alarm symptoms (Table 1)[25], malignancy of the gastrointestinal system, inflammatory bowel disease or other chronic diseases in other systems, metabolic or endocrine diseases (diabetes, hyperthyroidism and hypothyroidism, adrenocortical diseases), heart insufficiency, chronic obstructive pulmonary disease, hepatic cirrhosis, chronic renal insufficiency, severe depression or a history of persistent use of medications and tobocco were excluded from the analysis. All patients were examined using microscopic evaluation of the stool, a stool occult blood test, digestive stool analysis, hemogram, erythrocyte sedimentation rate, blood glucose, urea, creatinine, liver tests, thyroid hormones (free T4, TSH), anti-endomisium IgA and anti-gliadin IgG antibodies with upper gastrointestinal endoscopy (for excluding Celiac disease and other upper GIS disorders), abdominal ultrasonography and colonoscopy. During colonoscopy, terminal ileum was intubated and then two biopsy samples were taken from the cecum, ascending, transverse, descending, sigmoid colon and rectum.

**Table 1:** Alarm features considered potentially relevant in the diagnosis of organic disease as opposed to Irritable Bowel Syndrome

Rectal bleeding
Family history of colon cancer, inflammatory bowel disease, or celiac disease
Night-time symptoms (awakening the patient from sleep)
Weight loss
Signs of anemia
Abdominal mass

Biopsy specimens were sent to the pathology laboratory in 10% formol solution. All biopsy samples were evaluated by two pathologists who specialized in the gastrointestinal system. Paraffin blocks were cut into 5 μm thick sections and stained with hematoxylin-eosin (H&E) and Gomori's trichrome stain. A diagnosis of lymphocytic colitis required an increase in intraepithelial lymphocytes to more than 15 lymphocytes/100 epithelial cells, surface epithelial damage with increased lamina propria, plasma cells and absent or minimal crypt architectural disruption. For a diagnosis of collagenous colitis, an increase/irregularity in subepithelial collagen (> 10 μm) that typically trapped superficial capillaries was required as well as the other inflammatory changes seen in lymphocytic colitis [12]. Focal active colitis is the term used to describe focal neutrophilic infiltration of colonic crypts. It may consist of one focus in a single biopsy, or multiple foci [19].

The study protocol was approved by our Institutional (Umraniye Training and Research Hospital) Research Ethics Committee and informed consent was obtained from each subject.

Statistical analysis was performed using NCSS (Number Cruncher Statistical System) 2007 & PASS (Power Analysis and Sample Size) 2008 Statistical Software (Utah, USA), SPSS version 11.0 and Microsoft Excel. Statistical comparisons of findings between the IBS and control groups were performed by χ^2 ^or Fisher's exact test. Logistic regression was used to adjust for differences in age and gender between groups. A p < 0.05 was considered statistically significant.

## Results

A total of 378 patients were included in the study, 193 were female (51.1%), and the mean age of was 50.51 ± 14.72 for all patients (range 16-84). Two hundred and twenty six patients were in the IBS groups and their average age was 46.13 ± 14.16, 125 (55.3%) were female and 101 (44.7%) were male. In the control group of 152 patients, 84 were males (55.3%), 68 were females (44.7%) and the average age was 57.01 ± 13.07 years. There was a statistically significant difference in the age distributions of the IBS and control groups (P < 0.01). Additionally, a statistically significant difference in the gender distribution between the two groups was present (p = 0.044). Lymphocytic colitis was present in 3.1% (7/226) of IBS patients and 0% of controls (p < 0.05)(Table 2). Collagenous colitis was found neither patient group, nor control group.

**Table 2:** Demographic and pathological data of IBS and control patients

	IBS (n = 226)	Control (n = 152)	*p*
**^a^Age; Mean ± SD**	46.13 ± 14.16	57.01 ± 1.,07	0.001**
**^b^Gender (female, %)**	125 (55.3%)	68 (44.7%)	0.044*
**^b^Lymphoscytic colitis**	7 (3.1%)	0 (0%)	0.045*
**^b^Focal active colitis**	15 (6.6%)	0 (0%)	0.001**

^a ^Student t test ^b ^χ ^2 ^test

*p < 0.05

**p < 0.01

The IBS group of 226 patients was divided into 3 subgroups. 64 patients (28.3%) were in IBS-C, 90 patients (39.8%) were in IBS-M and 72 patients (31.9%) were in IBS-D. Serum biochemical tests, hemoglobin values and stool tests and macroscopic colonoscopic examinations were normal in all patients. MC was present in 4.32% (7/126) of non-constipated patients (diarrhea predominant and mixed) and in 3.1% of all IBS patients (7/226). However, lymphocytic colitis differed between non-constipated IBS group (IBS-D and IBS-M) and constipated IBS patients (p < 0.01). No lymphocytic colitis was seen in the constipation dominant group, only 1 case (1.1%) was seen in the mixed group and 6 cases (8.3%) were seen in the diarrhea dominant group (Table 3 and Table 4).

**Table 3:** Demographic and pathological data of IBS subgroups

	IBS subgroups			*p*
		
	IBS-C (n = 64)	IBS-M (n = 90)	IBS-D (n = 72)	
**^α^Age (Mean ± SD)**	43.54 ± 13.17	47.53 ± 13.54	46.68 ± 15.57	*0.211*
**^b^Gender (female,%)**	43 (67.2%)	45 (50%)	37 (51.4%)	*0.077*
**^β^Lymphochytic colitis n (%)**	0 (0%)	1 (1.1%)	6 (8.3%)	0.007**
**^β^Focal active colitis n (%)**	1 (1.6%)	10 (11.1%)	4 (5.6%)	*0.058*

^α ^Oneway Anova test

^β ^*χ ^2 ^test or Fisher's exact test*

**p < 0.01

**Table 4:** Demografic characteristics of patients with lymphocytic colitis and focal active colitis

	Age	Gender	IBS Subtype
**Lymphocytic colitis**	(Mean age: 60.5)		

**Patient 1**	49	Female	IBS-D
**Patient 2**	49	Female	IBS-M
**Patient 3**	56	Female	IBS-D
**Patient 4**	56	Female	IBS-D
**Patient 5**	69	Female	IBS-D
**Patient 6**	70	Female	IBS-D
**Patient 7**	75	Female	IBS-D

**Focal active colitis**	(Mean age: 45.7)		

**Patient 1**	21	Male	IBS-D
**Patient 2**	24	Male	IBS-M
**Patient 3**	30	Female	IBS-D
**Patient 4**	31	Female	IBS-M
**Patient 5**	32	Male	IBS-M
**Patient 6**	49	Male	IBS-M
**Patient 7**	50	Male	IBS-D
**Patient 8**	50	Female	IBS-M
**Patient 9**	51	Female	IBS-M
**Patient 10**	54	Male	IBS-M
**Patient 11**	54	Female	IBS-M
**Patient 12**	54	Male	IBS-M
**Patient 13**	60	Female	IBS-C
**Patient 14**	60	Male	IBS-M
**Patient 15**	66	Female	IBS-D

IBS-C: Constipated predominant, IBS-D: Diarrhea predominant, IBS-M:Mixed

Focal active colitis was found in 6.6% (15/226) of IBS patients and in none of the controls (p < 0.01). There was no significant difference among the incidence of focal active colitis according to IBS subtype (p > 0.05), although there was a higher incidence in the mixed group. Of the patients with FAC, 1.6% (1/64) were in the constipation predominant group, 11.1% (10/90) were in the alternate group and 5.6% (4/72) were in the diarrhea predominant group (Table 2).

## Discussion

IBS is a public health problem since it is widely seen and does not have a cure [26]. Some experts claim that LC is a precursor to CC [9-11]. A previously published study evaluated 89 patients suspected to have IBS and 59 controls using flexible sigmoidoscopy and rectal biopsy. These authors reported that no IBS patients or controls had macroscopic or microscopic findings that resulted in a change in diagnosis. Specifically, the authors identified no patients or controls with microscopic colitis. But according to recently published a study by Kao *et al*. in which a total of 547 cases of MC were examined, MC had a higher occurrence in IBS than in controls (P < 0.001)[27]. Conditions such as celiac disease, IBS, and thyroid disease were found to be related to MC. Furthermore, neither an increased risk of colorectal cancer nor IBD was associated with MC [27]. Chey *et al*. found that colonoscopy and colonic mucosal biopsies were able to identify an alternative diagnosis in 1.9% (9/466) of non-constipated IBS patients [28]. Of these nine patients, seven had microscopic colitis, one had Crohn's disease, and one had ulcerative colitis. The overall prevalence of microscopic colitis was found to be 1.5% in a large cohort of non-constipated IBS patients. In a subgroup analysis of IBS patients over the age of 45, the prevalence of microscopic colitis was 2.3% (4/171). In our study we found that all subjects with microscopic colitis were at least 49 years old and microscopic colitis was seen in 4.32% (7/126) of non-constipated IBS patients and in 3.1% of all IBS patients (7/226). All MC patients were over 45 years old and the average age was 62 ± 8. Limsui *et al*. found that 56% of the 131 patients diagnosed with microscopic colitis fulfilled the Rome III criteria for IBS and that 33% had been diagnosed with IBS before receiving the diagnosis of microscopic colitis. Therefore, patients with suspected diarrhea-predominant IBS should undergo biopsies of the colon to investigate possible microscopic colitis if symptoms are not well controlled by anti-diarrheal therapy [29].

A retrospective study found that 11% (43/376) of patients with LC and 18% (30/171) of patients with CC had been diagnosed with IBS before receiving the diagnosis of MC [27]. In two large studies of patients with MC, the mean age of diagnosis was found to be in the seventh decade [27,29]. A recent review on this topic suggested that between 18% and 34% of patients with collagenous colitis may not be identified if only rectosigmoid biopsies are obtained [30].

IBS has 3 subtypes according to its clinical course (constipation predominant, diarrhea predominant and mixed). IBS can be differentiated from MC only by histopathological investigation. Symptoms of MC are often attributed to diarrhea predominant IBS [31]. In view of this information, the present study explored if mucosal pathology, including MC, could be identified in IBS patients using microscopic evaluation, given that macroscopically, the colonic mucosa was normal.

Microscopic colitis is a rare disease [12-16]. However, Tuncer *et al*. reported that there is a 23.3% MC prevalence in IBS patients compared to a 5% prevalence in controls [32]. According to a study by Olesen *et al*., MC was diagnosed in 10% of all Swedish patients (1018 patients) with non-bloody diarrhea referred for colonoscopy. In the subset of patients older than 70, the prevalence was almost 20% [14]. Another study found that lymphocytic colitis was diagnosed in 199 cases. The female/male ratio was 2.4:1. The median age at diagnosis was 59 (range: 48-70) years. The most frequent symptoms were diarrhea (96%), abdominal pain (47%) and weight loss (41%). The symptom course was chronic intermittent in 30% of patients, chronic continuous in 7%, and single attack in 63%. MC patients may have constipation and there may be an association between MC and chronic constipation. Chronic constipation was found in 43.39% of MC patients in the study performed by Barta *et al*. [17,26]. However, in our study, MC was not found in the constipation predominant IBS subgroup. According to the American College of Gastroenterology Task Force [25], "routine colonic imaging is not recommended in patients younger than 50 years of age with typical IBS symptoms and no alarm features. When colonoscopy is performed in patients with IBS-D, obtaining random biopsies should be considered to rule out microscopic colitis."

Focal active colitis is characterized by focal crypt damage caused by neutrophils and may be associated with infections, ischemia, Crohn's disease, partially-treated ulcerative colitis and IBS [19]. There are reports that there is an association between focal active colitis and oral sodium phosphate ingestion [33-36]. Driman *et al*. indicated that evidence is emerging that sodium phosphate is a commonly used oral laxative agent, causes aphthoid ulcers and/or FAC in the colon and rectum. FAC was present in 11 of 316 patients (3.5%) who had biopsies but who were otherwise normal, as determined by endoscopic evaluation [33]. In a follow-up study by Xin *et al*., which consisted of 29 patients who were diagnosed with focal active colitis, the disease duration ranged from 4 months to 7 years with a mean of 4.2 years. Eight patients (27.6%) developed Crohn's disease. Pediatric patients with focal active colitis have a much higher incidence of Crohn's disease than adults. Hence, it is important to document the presence of focal active colitis in pediatric patients [21]. In this study we found that 6.6% of IBS patients had focal active colitis (15/226) and this ratio is higher than that reported in previous studies. It may be eligible take a routine biopsy in female patients and in patients over 50 years old.

We acknowledge the limitation of the study is the difference between the groups in terms of age and gender. The percentage of women with IBS is 55.3%. This percentage is close to the female:male ratio of previous studies from Turkey. The mean age of the control group was higher than the IBS patients. However, this situation was due to selection of asymptomatic patients screened for colorectal cancer and anemia.

## Conclusion

Microscopic colitis can be identified in patients with diarrhea predominant IBS and in women of older age with IBS. It appears reasonable to test for microscopic colitis in those patients by performing a colonic biopsy. Focal active colitis was found to be more common than expected. Routine biopsy of normal colonic mucosa helps identify rare miscellaneous causes of colitis.

## Abbreviations

CC: collagenous colitis; FAC: focal active colitis; IBS: irritable bowel syndrome; LC: lymphocytic colitis; MC: microscopic colitis

## Competing interests

The authors declare that they have no competing interests.

## Authors' contributions

AS participated in coordination and drafted the manuscript. KO conceived of the study, and participated in its design and coordination and helped to draft the manuscript. TC and AN helped collecting the data of the patient. RK conceived of the study and participated in its design and coordination and helped to draft the manuscript. UA has contributed to statistic analysis and manuscript preparation. HMS has contributed to study design and has coordinated research team. All authors read and approved the final manuscript.

## Pre-publication history

The pre-publication history for this paper can be accessed here:

http://www.biomedcentral.com/1471-230X/11/96/prepub

## References

[B1] Thompson WG Longstreth GF Drossman DA Heaton KW Irvine EJ Müller-Lissner SA Functional bowel disorders and functional abdominal pain Gut 1999 45 Suppl 2 II43 7 10457044 10.1136/gut.45.2008.ii43 PMC1766683

[B2] Karaman N Turkay C Yonem O Irritable bowel syndrome prevalence in city center of Sivas Turk J Gastroenterology 2003 14 2 128 31 14614640

[B3] Celebi S Acik Y Deveci SE Bahcecioglu IH Ayar A Demir A Durukan P Epidemiological features of irritable bowel syndrome in a Turkish urban society Journal of gastroenterology and hepatology 2004 19 7 738 43 10.1111/j.1440-1746.2004.03367.x 15209618

[B4] Hungin AP Whorwell PJ Tack J Mearin F The prevalence, patterns and impact of irritable bowel syndrome: an international survey of 40,000 subjects Alimentary pharmacology & therapeutics 2003 17 5 643 50 10.1046/j.1365-2036.2003.01456.x 12641512

[B5] Jones R Investigating lower bowel symptoms in general practice BMJ 1992 304 6841 1521 2 10.1136/bmj.304.6841.1521 1628048 PMC1882424

[B6] Talley NJ Spiller R Irritable bowel syndrome: a little understood organic bowel disease? Lancet 2002 360 9332 555 64 10.1016/S0140-6736(02)09712-X 12241674

[B7] Read NW Krejs GJ Read MG Santa Ana CA Morawski SG Fordtran JS Chronic diarrhea of unknown origin Gastroenterology 1980 78 2 264 71 7350049

[B8] Lindstrom CG 'Collagenous colitis' with watery diarrhoea--a new entity? Pathologia Europaea 1976 11 1 87 9 934705

[B9] Bowling TE Price AB al-Adnani M Fairclough PD Menzies-Gow N Silk DB Interchange between collagenous and lymphocytic colitis in severe disease with autoimmune associations requiring colectomy: a case report Gut 1996 38 5 788 91 10.1136/gut.38.5.788 8707130 PMC1383166

[B10] Jawhari A Talbot IC Microscopic, lymphocytic and collagenous colitis Histopathology 1996 29 2 101 10 10.1046/j.1365-2559.1996.d01-498.x 8872143

[B11] Mosnier JF Larvol L Barge J Dubois S De La Bigne G Hénin D Cerf M Lymphocytic and collagenous colitis: an immunohistochemical study The American journal of gastroenterology 1996 91 4 709 13 8677934

[B12] Pardi DS Smyrk TC Tremaine WJ Sandborn WJ Microscopic colitis: a review The American journal of gastroenterology 2002 97 4 794 802 10.1111/j.1572-0241.2002.05595.x 12003412

[B13] Fernández-Bañares F Salas A Forné M Esteve M Espinós J Viver JM Incidence of collagenous and lymphocytic colitis: a 5-year population-based study The American journal of gastroenterology 1999 94 2 418 23 10022639 10.1111/j.1572-0241.1999.00870.x

[B14] Olesen M Eriksson S Bohr J Järnerot G Tysk C Microscopic colitis: a common diarrhoeal disease. An epidemiological study in Orebro, Sweden, 1993-1998 Gut 2004 53 3 346 50 10.1136/gut.2003.014431 14960513 PMC1773978

[B15] Bohr J Tysk C Eriksson S Järnerot G Collagenous colitis in Orebro, Sweden, an epidemiological study 1984-1993 Gut 1995 37 3 394 7 10.1136/gut.37.3.394 7590436 PMC1382821

[B16] Olesen M Eriksson S Bohr J Järnerot G Tysk C Lymphocytic colitis: a retrospective clinical study of 199 Swedish patients Gut 2004 53 4 536 41 10.1136/gut.2003.023440 15016748 PMC1774011

[B17] Barta Z Mekkel G Csípo I Tóth L Szakáll S Szabó GG Bakó G Szegedi G Zeher M Microscopic colitis: a retrospective study of clinical presentation in 53 patients World journal of gastroenterology: WJG 2005 11 9 1351 5 15761974 10.3748/wjg.v11.i9.1351 PMC4250683

[B18] Leigh C Elahmady A Mitros FA Metcalf A al-Jurf A Collagenous colitis associated with chronic constipation The American journal of surgical pathology 1993 17 1 81 4 10.1097/00000478-199301000-00010 8447512

[B19] Greenson JK Stern RA Carpenter SL Barnett JL The clinical significance of focal active colitis Human pathology 1997 28 6 729 33 10.1016/S0046-8177(97)90183-0 9191008

[B20] Volk EE Shapiro BD Easley KA Goldblum JR The clinical significance of a biopsy-based diagnosis of focal active colitis: a clinicopathologic study of 31 cases Modern pathology: an official journal of the United States and Canadian Academy of Pathology, Inc 1998 11 8 789 94 9720510

[B21] Brown PI Greenson JK The clinical significance of focal active colitis in pediatric patients The American journal of surgical pathology 2003 27 8 1134 8 10.1097/00000478-200308000-00011 12883246

[B22] Saito YA Locke GR Talley NJ Zinsmeister AR Fett SL Melton LJ A comparison of the Rome and Manning criteria for case identification in epidemiological investigations of irritable bowel syndrome The American journal of gastroenterology 2000 95 10 2816 24 10.1111/j.1572-0241.2000.03192.x 11051354

[B23] Balboa A Mearin F Badía X Benavent J Caballero AM Domínguez-Muñoz JE Garrigues V Piqué JM Roset M Cucala M Figueras M Impact of upper digestive symptoms in patients with irritable bowel syndrome European journal of gastroenterology & hepatology 2006 18 12 1271 7 10.1097/01.meg.0000243870.41207.2f 17099375

[B24] Brandt LJ Chey WD Foxx-Orenstein AE Schiller LR Schoenfeld PS Spiegel BM Talley NJ Quigley EM An evidence-based position statement on the management of irritable bowel syndrome. American College of Gastroenterology Task Force on Irritable Bowel Syndrome The American journal of gastroenterology 104 Suppl 1 S1 35 10.1038/ajg.2008.122 19521341

[B25] Olden KW Diagnosis of irritable bowel syndrome Gastroenterology 2002 122 6 1701 14 10.1053/gast.2002.33741 12016433

[B26] Hahn B Watson M Yan S Gunput D Heuijerjans J Irritable bowel syndrome symptom patterns: frequency, duration, and severity Digestive diseases and sciences 1998 43 12 2715 8 10.1023/A:1026663613695 9881504

[B27] Kao KT Pedraza BA McClune AC Rios DA Mao YQ Zuch RH Kanter MH Wirio S Conteas CN Microscopic colitis: a large retrospective analysis from a health maintenance organization experience World journal of gastroenterology: WJG 2009 15 25 3122 7 10.3748/wjg.15.3122 19575491 PMC2705734

[B28] Chey WD Nojkov B Rubenstein JH Dobhan RR Greenson JK Cash BD The yield of colonoscopy in patients with non-constipated irritable bowel syndrome: results from a prospective, controlled US trial The American journal of gastroenterology 2010 105 4 859 65 10.1038/ajg.2010.55 20179696 PMC2887227

[B29] Limsui D Pardi DS Camilleri M Loftus EV Jr Kammer PP Tremaine WJ Sandborn WJ Symptomatic overlap between irritable bowel syndrome and microscopic colitis Inflammatory bowel diseases 2007 13 2 175 81 10.1002/ibd.20059 17206699

[B30] Yantiss RK Odze RD Optimal approach to obtaining mucosal biopsies for assessment of inflammatory disorders of the gastrointestinal tract The American journal of gastroenterology 2009 104 3 774 83 10.1038/ajg.2008.108 19209164

[B31] Loftus EV Microscopic colitis: epidemiology and treatment The American journal of gastroenterology 2003 98 12 Suppl S31 6 14697916 10.1016/j.amjgastroenterol.2003.10.008

[B32] Tunçer C Cindoruk M Dursun A Karakan T Prevalence of microscopic colitis in patients with symptoms suggesting irritable bowel syndrome Acta gastro-enterologica Belgica 2003 66 2 133 6 12891921

[B33] Driman DK Preiksaitis HK Colorectal inflammation and increased cell proliferation associated with oral sodium phosphate bowel preparation solution Human pathology 1998 29 9 972 8 10.1016/S0046-8177(98)90203-9 9744314

[B34] Lee FD Drug-related pathological lesions of the intestinal tract Histopathology 1994 25 4 303 8 10.1111/j.1365-2559.1994.tb01347.x 7835834

[B35] Wong NA Penman ID Campbell S Lessells AM Microscopic focal cryptitis associated with sodium phosphate bowel preparation Histopathology 2000 36 5 476 8 10866530

[B36] Zwas FR Cirillo NW el-Serag HB Eisen RN Colonic mucosal abnormalities associated with oral sodium phosphate solution Gastrointestinal endoscopy 1996 43 5 463 6 10.1016/S0016-5107(96)70286-9 8726758

